# Supplementary material for: Evaluating the state-of-the-art in mapping research spaces: A Brazilian case study
Source: PLoS One. 2021 Mar 18;16(3):e0248724. doi: 10.1371/journal.pone.0248724 (PMC7971485; doi:10.1371/journal.pone.0248724)
Supplement: Attachment — Submitted filename: comments.pdf [file pone.0248724.s001.pdf]

Although I am not an expert in this field, I thought the article “Evaluating the state-of-the-art in mapping research spaces: a Brazilian case study” was very well written. Congratulations!

My main comments are:

1) There is a relatively large number of seemingly arbitrary decision made by the authors when summarizing their data. For instance, the calculation of $X_{sf}(t)$ and $P_{sf}(t)$ seem arbitrary. Why set theta to 0.1, aside from the fact that a past study has set theta to this number? What is the impact of using $\sum_p \frac{1}{n_p m_p}$ to calculate $X_{sf}(t)$? Does this end up downplaying researchers that tend to work as part of large teams? While the authors justify several of these decisions by pointing to earlier articles, to me it is still unsettling that many of these decisions may fundamentally change the results the authors found.

2) In the section “Mapping publication data from Lattes to venues and their fields in Scopus”, the authors describe how a relatively small fraction of papers were matched. How common is this problem in the field? While the authors described well why this occurs, it would be useful to show if past studies have faced similar problems and compare, if possible, the proportion of match they obtained with those from past studies. This could help dispel the fear that this low proportion of matches is anomalous in the field, potentially jeopardizing all the downstream results.

Here are some additional but minor comments:

1) The only section that I found harder to follow was the section “Impact of institution characteristics on prediction quality”. The authors should try to write this section in such a way that it is more straight-forward to understand for scientists in other fields.

2) "The impact of associating work done by a researcher in a former institution or geographical state to the current one is small, since its effect is diluted when computing the RCA for the institution/state." Can you better describe why this impact would be small? This is not clear to me.

3) The matrix $\phi_{ff'}^{(freq)}(t)$ is not symmetric given that the denominator is the number of scientists that published in f’. As a result, it is not clear why it can be interpreted as the strength of the links between research fields.

4) For the embedding model, it is not clear how the set $\mathbf{B}_s(\boldsymbol{t})$ is related to the cosine similarity $S_{ff'}(t)$. Please describe this model more thoroughly

5) “The fact that the average number of publications per year grows”: this could be due to the increase of articles with more co-authors given that the same paper is counted multiple times if it shows up in multiple Lattes profiles

6) It is impossible to read text in Fig. 12. The image resolution is too low.
