# Supplementary material for: Evaluating the state-of-the-art in mapping research spaces: A Brazilian case study
Source: PLoS One. 2021 Mar 18;16(3):e0248724. doi: 10.1371/journal.pone.0248724 (PMC7971485; doi:10.1371/journal.pone.0248724)
Supplement: Attachment — Submitted filename: Response to Reviewers PLOS ONE - Rev1.pdf [file pone.0248724.s002.pdf]

Response to the Reviewers

We would like to thank the reviewers for their feedback, which allowed us to greatly improve our work. We believe we have addressed all concerns raised by the reviewers appropriately. Please find below our detailed response to the reviewers.

Reviewer #1:

R1.1: The authors describe an interesting field of connecting Research Areas and mapping them. The paper is well written and provides interesting Information how science works in a defined area. However, the paper is very long and should be focussed on its main statement.

Authors' response: We agree with the reviewer that some sections of the manuscript were deviating from the main statement or excessively long. Therefore, we have removed the section regarding the impact of the vector dimension in the Embedding Model and shortened the section on the analysis of individual predictions for scientists so as to include only two (instead of three) researchers: one for which the model performs well and another for which it presents issues.

R1.2: The abstract should be revised and the citations (Guervarra et al., Chinazzi et al, etc.) should be deleted or transfered to Introduction. Readers who aren't expert of this theme would be distracted from the main focus of this work.

Authors' response: As per the reviewer's suggestions, we moved the citations to the introduction. The abstract is indeed targeted towards a more general audience now.

R1.3: Please describe the abbreviations sufficiently (DoD, UCSD, word2vec, NEET).

Authors' response: We thank the reviewer for pointing out that the descriptions of some abbreviations were missing in the previous submission. We have included them in the revised version.

R1.4: Could the authors provide more information regarding scientists with a research stay abroad. Are These works integrated in this model? What happens if a scientist changes her/his name after marriage? Are these changes considered in the methodology?

Authors' response: Researchers that are resident in Brazil have incentives to keep their CVs up-to-date and complete in the Lattes Platform, since including past publications can confer advantages when competing for research grants, scholarships and awards. Hence, papers published by a researcher during stays abroad are very likely to be included in the data, unless he/she decided not to return to Brazil afterwards.

Name changes after marriage/divorce are not an issue because the researcher adds his/her own publications on the profile (either by DOI or by filling out a form). Thus, the data contains the researcher's papers despite possible name changes.

Reviewer #2: In this manuscript, a comparative analysis of two methods to evaluate the ability of them to predict the potential of some researchers to do contribution in other fields.

R2.1: The idea presented is interesting, but I don't know the necessity to perform this comparison. Moreover, authors don't take into account that the fields are not disjoint. That is, some publications are classified into two or more fields. For example, Scientometrics could be seen as computer science, and library and information science, among other disciplines. For example, the journal of Scientometrics, is indexed in two categories. So, researchers publishing in this journal have presence in both areas. This issue is important, and could affect the main hypothesis of the paper.

Authors' response: In the past few years, several efforts have been made towards understanding how scientific fields are connected and how they are changing. This line of research is likely to grow as science is under continuous evolution. We believe the comparison presented in our work is not only long due, but also useful as a starting point for subsequent works on research maps. Specifically, it shows that the Frequentist model outperforms the Embedding model when predicting transitions for individuals and sets new methodological standards for future comparisons.

*It is possible that the Methodology section in the previous submission was causing the impression that each venue in the Scopus database is categorized as belonging to one and only one specific field (within one intermediate classification which was, in turn, in one macro area). We changed this section to make clear that each venue can be associated with multiple fields. A detailed explanation of how this is accounted for by the models can be found in our answer to comment **R3.1**.*

R2.2: I miss references and comments regarding science mapping analysis and bibliographical networks. Authors should cite too small when they talk about co-citation, and do the same with coupling and other networks.

There are more ways to create a map, for example, based on author information, that is, a co-authors networks (author name, or affiliation).

Authors' response: The reviewer is correct in stating that methods based on co-authorship networks were not adequately covered in the Related Works section. These techniques are of paramount importance in the analysis of scientific collaboration and academic productivity. Although, to the best of our knowledge, they have not been used for creating research maps, we included a paragraph in the revised manuscript listing relevant studies and applications, as

well as some intuition on how co-authorship networks could be used in the future to understand how scientific fields are connected:

"A related approach, based on paper co-authorship, has been used mainly for understanding scientific interactions. The properties and dynamics of co-authorship networks have been well studied [10,11], and these networks were shown to be especially useful for future performance prediction [12,13,14]. They have also been used for measuring the strength of the relationship between groups (e.g., countries [15]). In principle, such networks could be used to create research maps by analyzing how researchers from different fields collaborate and even to make predictions regarding the likelihood that an individual becomes active in one field based on past interactions. However, they have not been used for this purpose, to the best of our knowledge."

[10] Barabási AL, Jeong H, Néda Z, Ravasz E, Schubert A, Vicsek T. Evolution of the social network of scientific collaborations. Physica A: Statistical Mechanics and its Applications. 2002;311(3):590–614. doi:https://doi.org/10.1016/S0378-4371(02)00736-7.7.

[11] Newman MEJ. In: Ben-Naim E, Frauenfelder H, Toroczkai Z, editors. Who Is the Best Connected Scientist? A Study of Scientific Coauthorship Networks. Berlin, Heidelberg: Springer Berlin Heidelberg; 2004. p. 337–370.

[12] .Abbasi A, Chung KSK, Hossain L. Egocentric analysis of co-authorship network structure, position and performance. Information Processing Management.7202012;48(4):671–679. doi:https://doi.org/10.1016/j.ipm.2011.09.001.

[13] Beaver D, Rosen R. Studies in scientific collaboration. Scientometrics. 26 Sep 1978;1(1):65 – 84. doi:10.1007/bf02016840.

[14] Parish AJ, Boyack KW, Ioannidis JPA. Dynamics of co-authorship and productivity across different fields of scientific research. PLOS ONE. 2018;13:e0189742. doi:10.1371/journal.pone.0189742.72615.

[15] Glänzel W. National characteristics in international scientific co-authorship relations. Scientometrics. 26 Apr 2001;51(1):69 – 115. doi:10.1023/a:1010512628145.

R2.3: The dataset employed is old, and not accurate. Authors only match 52% of the publications. Maybe it could e better use directly Scopus as database, and join by Scopus author ID.

Authors' response: The dataset used in the paper was collected in early 2017, thus we use all papers published up to 2016. Since then, the Lattes Platform has put in place new mechanisms to obstruct crawlers, preventing us from gathering a more up-to-date dataset. As a reference for assessing the recency of the data, the ones used by Guevara et al. (2016) and Chinazzi et al. (2019) considered data up to 2014 and 2009, respectively. Nevertheless, we have shown that

the results are robust to the period under analysis (please refer to the "Second experimental setup" section) and our conclusions should hold for the most recent data.

We agree with the reviewer that the fact that we could only match 52% of the publications could be a matter of concern. Therefore, we set out to investigate what was causing the failures in the matching process. Our main hypothesis was that many of these works were published in Portuguese (in venues that have Brazil as the main target audience). We used Google Translate's API to identify the language in which the venues' names were written. Next, for each of the languages, we computed the fraction of the records that were matched to a venue using the Scopus database. The results are shown in Table 1.

Detected language L	*Fraction f_L of total records*	*Fraction of records in f_L successfully matched*
English	*47.6%*	*76.1%*
Portuguese	*42.1%*	*28.3%*
Spanish	*5.9%*	*29.1%*
Other languages	*4,4%*	*39%*

Table 1: Statistics of the success ratios when matching venue titles to Scopus by language

These results corroborate our hypothesis: 62.3% of the non-successful matchings correspond to papers published in Portuguese-language venues. Furthermore, the coverage for English records is 76.1%.

We then investigated whether the low matching rate for venue titles in Portuguese could be due to typos, diacritical marks, or name variants associated with the same venue. To do so, we randomly sampled 80 non-successful matchings from that subset and manually tried to find them in the Scopus database using substrings and spelling variations. Out of 80 samples, we found only 2 matches (2.5%):

- *Lattes: Boletim de Geociências* **da** *Petrobrás / Scopus: Boletim de Geociências* **-** *Petrobrás*
- *Lattes: Revista* **de** *Neurociências / Scopus: Revista Neurociências*

While we could remove "de"/"da"/"do" (in English, "of") from venue titles in Portuguese, this could incur in false positives: e.g., "Revista de Neurociências" (Journal of Neurosciences) and "Revista Neurociências" (Neurosciences Journal) could as well be two different journals. Moreover, such change would not improve the number of matches significantly.

In sum, we found that (i) most of the non-successful matchings occur because the listed venues are not present in the Scopus database and that (ii) venues in English match at 76.1% success rate. Considering that the publications written in Portuguese have a limited reach, we believe that disregarding these non-successful matchings should not significantly impact our results on how scientific fields are organized and how they are evolving.

R2.4: The figures have a very low quality. Very low.

Authors' response: We apologize as the PDF generated by the submission system apparently has distorted the TIF figures attached to the manuscript. The problem seems to be solved in the revised submission, which uses EPS files instead.

Reviewer #3: This article is very well written. However, I do have multiple comments that I believe can help improve it. These are described in detail in the attached document.

Authors' response: We are very thankful for the detailed feedback. The suggestions have been incorporated to the text, improving its readability and the methodology used in the evaluation.

My main comments are:

R3.1: There is a relatively large number of seemingly arbitrary decision made by the authors when summarizing their data. For instance, the calculation of $Xsf(t)$ and $Psf(t)$ seem arbitrary. Why set theta to 0.1, aside from the fact that a past study has set theta to this number? What is the impact of using $\sum_p 1/n_p m_p$ t to calculate $Xsf(t)$? Does this end up downplaying researchers that tend to work as part of large teams? While the authors justify several of these decisions by pointing to earlier articles, to me it is still unsettling that many of these decisions may fundamentally change the results the authors found.

Authors' response: The reviewer raises a valid concern regarding the modeling decisions in the manuscript. We completely agree that it is worth discussing or even studying their impact.

For instance, the definition of matrix X(t) controls for the number of authors n_p and the number of fields m_p associated with the paper's venue. Ignoring one of those factors would give more weight to publications with more authors or to venues that cover multiple fields (e.g., Scientometrics). Another way of discounting these factors would be normalizing by log-scaled values. Both of these alternatives would result in more transitions and would prevent us from comparing the results with the existing literature. We added a footnote to discuss this matter.

On the other hand, we believe it is reasonable to consider the threshold θ, used for defining the presence matriz P(t), a tunable hyperparameter. Inspired by the reviewer's question, we investigated the impact of θ on the prediction accuracy, by setting it to values in {0.025, 0.05, 0.10, 0.20, 0.40}. As expected, larger values make the transitions less likely to occur, as it becomes "harder" to be active in a field.

	Frequentist Model		***Embedding Model***	
Threshold θ	*Average AUC*	*Median AUC*	*Average AUC*	*Median AUC*
0.025	*0.87758*	*0.90903*	*0.84478*	*0.88688*
0.05	*0.87859*	*0.90986*	*0.85145*	*0.89640*

0.10	*0.87936*	*0.90975*	*0.83309*	*0.87919*
0.20	*0.87913*	*0.90839*	*0.84307*	*0.88438*
0.40	*0.87401*	*0.90177*	*0.83556*	*0.87199*

Table 2: Prediction results for scientists' transition Inactive → Active for various thresholds

	Frequentist Model		***Embedding Model***	
Threshold θ	*Average AUC*	*Median AUC*	*Average AUC*	*Median AUC*
0.025	*0.85589*	*0.88279*	*0.82594*	*0.86215*
0.05	*0.85593*	*0.88294*	*0.83234*	*0.86565*
0.10	*0.85538*	*0.88311*	*0.81540*	*0.84955*
0.20	*0.84791*	*0.87397*	*0.81599*	*0.84678*
0.40	*0.82658*	*0.85166*	*0.79819*	*0.82429*

Table 3: Prediction results for institutions' transition Inactive → Active for various thresholds

Interestingly, the area under the ROC curve remains relatively stable across the different values of θ. Even in the case of institutions' transition Inactive to Active, where the variation is larger, the average AUC varies at most 4% (Embedding Model). This indicates that the models are robust to the choice of the threshold value. Yet, there seems to be a "sweet spot" around θ=0.05, as both models achieved peak performance around this value. For this reason, we decided to re-run all the prediction experiments with this new threshold and report them in the revised submission, as they are more representative of the models under evaluation. This procedure is briefly described in the "First experimental setup" section.

The experiments in the case study section were kept with θ=0.10, since the resulting network was more suitable for the analysis, as explained in the revised paragraph excerpt:

"Differently than in Jaffe et al. (2020), our graph depends on a threshold θ used for defining the presence matrix. Note that there is an interplay between θ and α, but there is no way to automatically tune them when conducting a qualitative analysis of the resulting backbone network. Hence, we perform experiments with different values of thresholds, ultimately setting θ=0.10 and α=0.20 (which is higher than the α= 0.05 value used in Jaffe et al. (2020)) since, in our case, these values yield networks that are neither too dense or too disconnected to interpret."

R3.2: In the section "Mapping publication data from Lattes to venues and their fields in Scopus", the authors describe how a relatively small fraction of papers were matched. How common is this problem in the field? While the authors described well why this

occurs, it would be useful to show if past studies have faced similar problems and compare, if possible, the proportion of match they obtained with those from past studies. This could help dispel the fear that this low proportion of matches is anomalous in the field, potentially jeopardizing all the downstream results.

Authors' response: This is a very good point and it was also raised by Reviewer 2. We have discussed this issue with Prof. Alberto H. F. Laender, the colleagues that provided us the Lattes dataset, who has also directed several researches that made use of it. Although none of his previous works attempted to match the papers' venues to the Scopus database, he pointed out that the 52% coverage rate was well within his expectations and that most of the non-successful matchings were likely to be publications in Portuguese, appearing in national venues. We were able to corroborate this hypothesis by conducting a separate study, which is described in the answer to comment ***R2.3****.*

Here are some additional but minor comments:

R3.3: The only section that I found harder to follow was the section "Impact of institution characteristics on prediction quality". The authors should try to write this section in such a way that it is more straight-forward to understand for scientists in other fields.

Authors' response: We agree with the reviewer that the way this section was not very clear or very readable in the previous submission. To begin with, the term "institution characteristics" was very imprecise and likely to be interpreted as aspects unrelated to the publication records. In addition to changing the section title to "Impact of statistics of the data associated with an institution on prediction quality", we made a series of changes to the text. In the revised submission, we start by clearly stating which statistics -- number of fields and papers -- are going to be analyzed, why and how. Towards the end of the section, we also summarize the conclusions from Figures 8 and 9.

R3.4: "The impact of associating work done by a researcher in a former institution or geographical state to the current one is small, since its effect is diluted when computing the RCA for the institution/state." Can you better describe why this impact would be small? This is not clear to me.

Authors' response: Here we are relying on the premise that, in Brazil, the bulk of a non-junior researcher's work is published under his/her most recent affiliation. We argue that this is the case because most of the country's research is conducted at public universities or national institutes and the career system designed by the government makes it very hard for an experienced researcher to move to another institution while keeping his/her current rank. Reproducing the case study for other countries would indeed require a more precise mapping between papers and affiliations at the time of publication. We have revised the entire paragraph to make this point more clear.

R3.5: The matrix $\phi(freq)(t)$ is not symmetric given that the denominator is the number of scientists ff' that published in f'. As a result, it is not clear why it can be interpreted as the strength of the links between research fields.

Authors' response: The reviewer is correct in that "strength" does not convey an asymmetric interpretation. We have modified the corresponding text so as to refer to $\phi(freq)(t)$ *as "flow".*

R3.6: For the embedding model, it is not clear how the set $\mathbf{B}s(t)$ is related to the cosine similarity $Sff'(t)$. Please describe this model more thoroughly

Authors' response: We thank the reviewer for pointing out that this part of the Embedding Model description lacked cohesion. In the revised version, after introducing the set $B_s(t)$ *of fields in which scientist s is active, we introduce the vector representations (a.k.a. embeddings)* vec_f^t *and explain that they are learned by an optimization function based on the similarity between vectors. Last, we explain that the similarity between vectors is measured by the cosine similarity.*

R3.7: "The fact that the average number of publications per year grows": this could be due to the increase of articles with more co-authors given that the same paper is counted multiple times if it shows up in multiple Lattes profiles

Authors' response: We certainly agree with the point raised by the reviewer. We have changed the text to clearly indicate that we are referring to the number of publication records.

A rigorous way to determine the growth in the number of publications would require us to identify which records correspond to the same paper (using a hashing technique based on paper title and author last names, for instance) and is out of the scope of the paper. Although we did not include it in the paper, we computed the number of yearly publications normalized by the number of authors, in response to the reviewer's comment. Figure 1 shows the results, suggesting that the number of unique papers is indeed increasing. The slight decline on the right hand side of the plot is likely due to right-censoring effects.

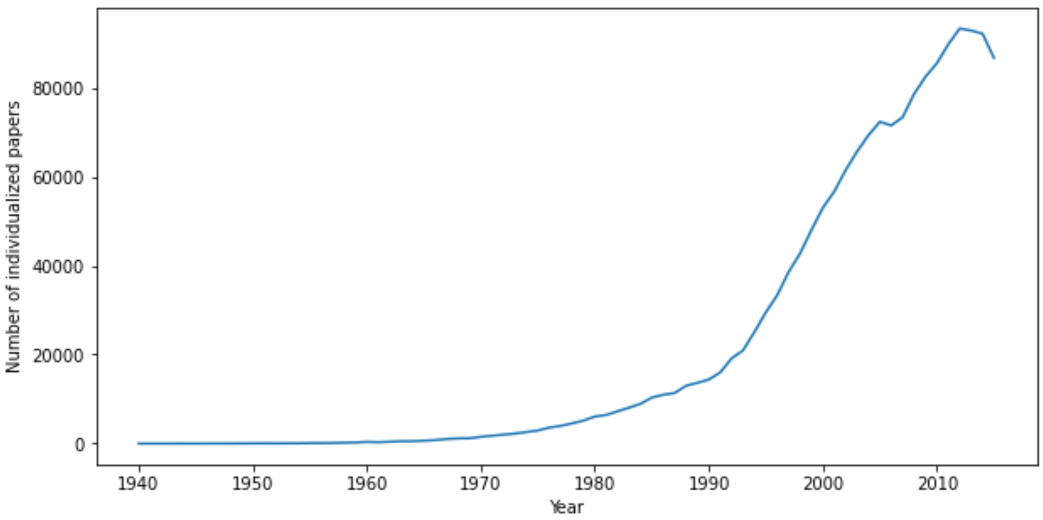

Figure 1: Yearly number of papers normalized by the number of authors

R3.8: It is impossible to read text in Fig. 12. The image resolution is too low.

Authors' response: We apologize as the PDF generated by the submission system apparently has distorted the TIF figures attached to the manuscript. The problem seems to be solved in the revised submission, which uses EPS files instead.
